# UHPLC Analysis of Saffron (*Crocus sativus* L.): Optimization of Separation Using Chemometrics and Detection of Minor Crocetin Esters

**DOI:** 10.3390/molecules23081851

**Published:** 2018-07-25

**Authors:** Angelo Antonio D’Archivio, Francesca Di Donato, Martina Foschi, Maria Anna Maggi, Fabrizio Ruggieri

**Affiliations:** 1 Dipartimento di Scienze Fisiche e Chimiche, Università degli Studi dell’Aquila, Via Vetoio, 67100 L’Aquila, Italy; didonatofrancesc@gmail.com (F.D.D.); martina.foschi.mf@gmail.com (M.F.); fabrizio.ruggieri@univaq.it (F.R.); 2 Hortus Novus srl, Viale Aldo Moro 28 D, 67100 L’Aquila, Italy; maria.magg@tiscali.it

**Keywords:** saffron, crocins, UHPLC analysis, separation optimisation, artificial neural network, response surface methodology

## Abstract

Ultra-high performance liquid chromatography (UHPLC) coupled with diode array detection (DAD) was applied to improve separation and detection of mono- and bis-glucosyl esters of crocetin (crocins), the main red-colored constituents of saffron (*Crocus sativus* L.), and other polar components. Response surface methodology (RSM) was used to optimise the chromatographic resolution on the Kinetex C18 (Phenomenex) column taking into account of the combined effect of the column temperature, the eluent flow rate and the slope of a linear eluent concentration gradient. A three-level full-factorial design of experiments was adopted to identify suitable combinations of the above factors. The influence of the separation conditions on the resolutions of 22 adjacent peaks was simultaneously modelled by a multi-layer artificial neural network (ANN) in which a bit string representation was used to identify the target analytes. The chromatogram collected under the optimal separation conditions revealed a higher number of crocetin esters than those already characterised by means of mass-spectrometry data and usually detected by HPLC. Ultra-high performance liquid chromatography analyses carried out on the novel Luna Omega Polar C18 (Phenomenex) column confirmed the large number of crocetin derivatives. Further work is in progress to acquire mass-spectrometry data and to clarify the chemical structure to the newly found saffron components.

## 1. Introduction

Saffron, the dried stigmas of *Crocus sativus* L., is a precious spice used worldwide as food additive for its coloring and flavoring properties. Besides culinary uses, saffron has been considered a natural remedy in traditional medicine since ancient times and is nowadays the growing subject of biomedical research aimed at investigating bio-activity of its ingredients [1,2]. Crocins, a family of water-soluble mono- and di-glycosyl esters of the polyene dicarboxylic acid crocetin, are the main constituents responsible for the appreciated saffron color [3,4]. Safranal (a monoterpene aldehyde) and picrocrocin (glycoside of safranal) are other two major saffron constituents, that mainly contribute to the aroma and bitter taste, respectively. High-performance liquid-chromatography (HPLC) with diode-array (DAD) or mass-spectrometry (MS) detection has been extensively applied to identify and quantify the water-soluble saffron components [3,4,5,6,7,8,9,10] for both quality control and geographical traceability purposes. Compounds structurally related to picrocrocin and flavonoids, kaempferol derivatives in particular, can also be determined by HPLC analysis of aqueous or hydro-alcoholic extracts [4,9,10,11,12]. To date, the chemical structure of 16 crocins has been identified by MS data [4,5,6]. These differ in the sugar moieties, glucoside (g), gentiobioside (G), neapolitanoside (n) or triglucoside (t), and in the *cis* and all-*trans* isomeric forms of crocetin. However, only the major crocins (between six and ten compounds) providing relatively intense chromatographic peaks can be easily detected in HPLC saffron characterization [3,5,7,8,9,10]. In particular, *trans*-crocetin bis(β-d-gentiobiosyl) ester, *trans*-crocetin (β-d-gentiobiosyl)(β-d-glucosyl) ester and *cis*-crocetin (β-d-gentiobiosyl)(β-d-glucosyl) ester, which account for more than 95% of the total crocins and few other crocetin derivatives dominate the observed chromatograms. Mass-spectrometry detection, although essential for collecting structural information on the saffron constituents, does not permit to highlight additional crocins as compared to those revealed by DAD under similar separation conditions [4,5,6,7]. Recently, more than 20 crocins have been identified in the HPLC-DAD analysis of Italian and Iranian saffron [13,14] and some of the minor crocetin derivatives newly found resulted to be powerful markers for the determination of saffron origin. However, geographical differentiation based on chemometric treatment of the HPLC chromatograms [13] can be less efficient under the condition of inadequate separation because of a partial loss of information regarding the saffron composition. Ultra-high performance liquid chromatography (UHPLC), in spite of faster analysis and higher separation efficiency than conventional HPLC, has been rarely applied before to saffron characterization [15,16,17]. The main aim of this investigation is to enhance separation of the polar saffron constituents, crocins in particular, by means of UHPLC-DAD under gradient elution to improve information on the qualitative chemical composition of aqueous extracts. The simultaneous and interactive influence of the mobile phase flow rate, the slope of a linear eluent gradient and the column temperature on the chromatogram resolution was investigated by response surface methodology (RSM). A full-factorial design of experiments (DOE) was used to identify appropriate combinations of the above factors. The response surface describing the UHPLC resolution was generated by combining the outputs of artificial neural network (ANN) trained to model the resolutions of adjacent peak pairs in the chromatogram. The UHPLC-DAD analysis carried out on the Kinetex C18 (Phenomenex, Torrance, CA, USA) column under the optimized separation conditions allowed the identification of new crocetin derivatives. The unexpectedly large number of crocins was confirmed by UHPLC analysis carried out on the Luna Omega Polar C18 (Phenomenex) column packed with a novel stationary phase having a polar modified surface. Preliminary mass-spectrum data support the attribution of the newly found saffron components to the family of crocetin esters and further work is in progress to clarify their chemical structure. 

## 2. Results and Discussion

### 2.1. Artificial Neural Network-Based Modeling of Chromatogram Resolution

#### 2.1.1. Artificial Neural Network Modeling of Peak Pair Resolution

**Figure 1:**
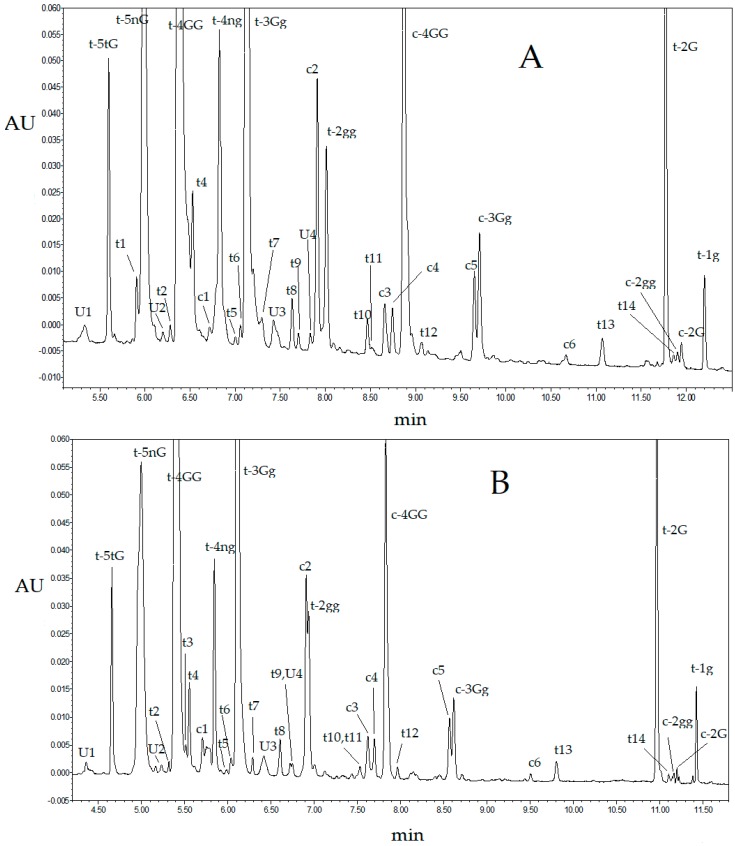
Ultra high-performance liquid-chromatography-diode-array (UHPLC-DAD) chromatograms detected at 440 nm of a saffron extract obtained with the column Kinetex C18 at (**A**) t_g_ = 10 min, T = 25 °C and (**B**) φ = 0.60 mL/min and t_g_ = 10 min, T = 25 °C and φ = 1.00 mL/min. Peak assignments are reported in Table 1.

Artificial neural networks were previously used in chromatography to handle complex regression problems, including RSM optimization [18,19,20] and retention prediction [21,22,23,24,25]. In this work, ANN multivariate modeling was applied to investigate the influence of the mobile phase flow rate (φ), the duration of a linear eluent gradient (t_g_) and the column temperature (T) on the chromatogram resolution of saffron extracts. The chromatograms observed for the combinations of the above factors defined according to a full-factorial DOE were considered to build the ANN-based model, while additional eight chromatograms were acquired for validation (Section 3.6.1). Figure 1 displays some representative UHPLC chromatograms, detected at 440 nm, namely the absorption maximum of crocins. Most of the observed peaks can be safely assigned to *trans*- or *cis*-crocins (Table 1) according to the spectral features (Section 3.5). We generated a single ANN model able to process simultaneously all the data related to the resolution of adjacent peaks observed in the chromatogram, using a bit string to represent each analyte pair. A similar strategy was previously adopted to optimize the gas-chromatographic separation of chlorinated pollutants [20] or predict the HPLC retention times of biologically active solutes under multilinear gradient elution conditions [26]. In addition to the three neurons associated with φ, t_g_ and T, 22 neurons corresponding to the chromatographic peaks of interest (shown in Table 1), ordered according to the retention time, were included in the ANN input layer. To link a given pair resolution R_ij_ with the corresponding couple of saffron metabolites ij, all these inputs were set to 0 with the exception of the ith and jth, which were 1. It follows that the network was called to process 25 input variables, three of them describing the separation system and the remaining 22 associated with the analyte pairs.

As 21 resolutions were measured for each point of the DOE, 21∙29 = 609 data samples were available for ANN calibration and 21∙8 = 168 for external prediction. Kennard-Stone algorithm [27] was applied to the calibration data after variable auto-scaling to design a training set (486 data samples) to be used in the ANN learning. The remaining 123 data samples were used in the ANN-based model validation to select the best combination of network architecture, activation function and learning duration. 

**Figure 2:**
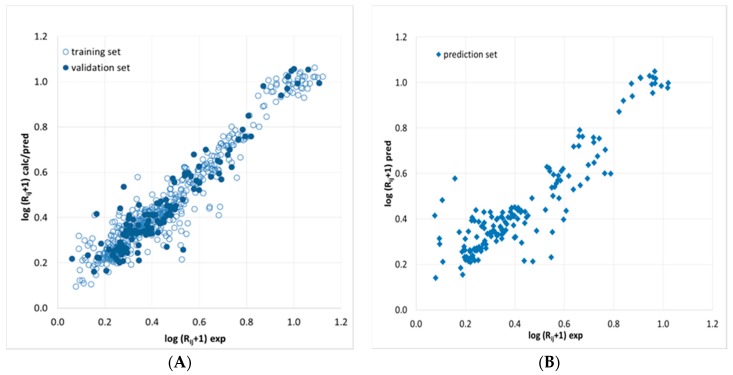
Agreement between (**A**) experimental (exp) resolutions and (**B**) calculated or predicted (calc/pred) responses of the ANN-based model. R_ij_, resolution of peak pairs.

Ultra high-performance liquid-chromatography separation of the saffron water-soluble colored constituents was optimized by analyzing a sample produced in L’Aquila (Abruzzo, Italy) in 2015 under the various experimental conditions defined by the selected DOE and in the additional data points designed for the external validation. Repeated analyses of a same extract kept in the auto sampler of the UHPLC apparatus revealed that peak areas of saffron metabolites did not change appreciably within 24 h from extraction. In any case, to avoid degradation of the target analytes, the UHPLC analyses were carried out on daily extracted samples. Many alternative networks were trained to predict the resolution of consecutive peak pairs R_ij_ (j = i + 1) in the observed chromatograms detected at 440 nm. The optimal network finally selected was the one providing the lowest validation error. In the training procedure, the updating of the initial weights, randomly generated within the range (−0.1, 0.1), was conducted until the validation started to increase. To ensure that the best model was not generated by a particular combination of the initial weights, the optimal network was re-trained 100 times and the outputs were averaged. Rather than the pair-resolution R_ij_, log(R_ij_ + 1) was chosen as the network response, because this transformation provided a more homogenous error distribution. The best validation performance was obtained with a 25-8-1 network having a hyperbolic tangent activation function in the hidden layer and learned for 65 epochs. Determination coefficients in training, validation and external prediction were 0.922, 0.893 and 0.823, respectively, while the related standard errors were 0.065, 0.071 and 0.097. The above statistical parameters suggest a good model, which is confirmed by the agreement between computed/predicted and target responses (Figure 2) showing a random distribution near the ideal line with the exception of a limited number of data samples that are modeled worse, but these points do not refer to specific solutes or experimental conditions.

Since ANN modeling does not provide a fitting equation, interpretation of the found model is not straightforward. To overcome this limitation, we applied the partial derivative method [28] that seeks to assess the sensitivity of the network output against slight changes in input variables. This procedure revealed that all the three factors related with the separation system, T, φ and t_g_, influence in a similar way the chromatographic resolution but the column temperature slightly prevails over the other two factors. The ANN-based model was also evaluated by sum of ranking differences (SRD) developed by Héberger [29] for ranking and comparison of methods and models. In particular, the ANN residuals were handled by SRD to compare the model performance in the various points of the DOE. Sum of ranking differences was computed by summing the absolute differences of data rankings with respect to the central point of the DOE, used as reference. The SRD analysis was validated using comparison of ranks by random numbers. The calculated SRD values associated to all the points of the DOE resulted to fall within the 95% confidence interval of the fitted Gaussian curve. It follows that the network ability to model the resolution of close peak pairs in the various experimental conditions defined by the DOE is substantially the same. 

#### 2.1.2. Generation of Surface Response for Global Resolution

**Figure 3:**
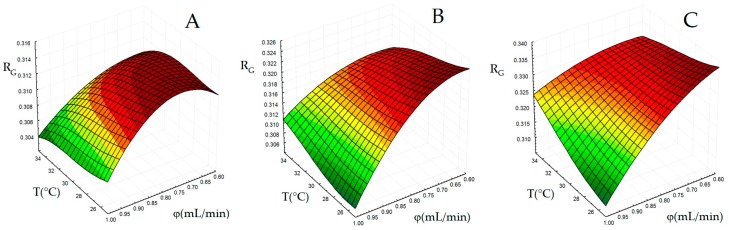
Plots of response surface for global resolution (R_G_) as a function of column temperature (T) and eluent flow rate (φ), the eluent gradient duration (t_g_) kept fixed at 8 (**A**), 10 (**B**) or 12 min (**C**).

Finding an adequate chromatographic separation of the components of complex mixtures is a multi-response optimization problem [30]. Ideally, the separation conditions should be set in order to separate and detect as many analytes as possible. However, since the changes in the separation variables do not influence in a similar way the overlapping degree of close peaks in different regions of the chromatogram, a compromise solution must be found. In this work, to transform the multi-response ANN output into a single response, we defined a global resolution (R_G_) parameter, as the medium of log(R_ij_ + 1) values. For each experimental condition, rather than including all the 21 predicted responses in the computation of R_G_, only a subset was considered. In particular, we excluded those analyte pairs providing either well separated or strongly overlapped peaks with an associated log(R_ij_ + 1) value poorly dependent on the experimental conditions. The pairs 4-5, 5-6, 7-8, 9-10, 11-12, 12-13, 13-14, 14-15, 15-16 and 21-22 were finally retained. Figure 3 shows the three-dimensional plots of R_G_ as function of T and φ for t_g_ fixed at 0.6, 0.8 and 1.0 min. 

Generalization ability of the response surface for R_G_ was indirectly tested by previous external validation of the ANN-based model, which permitted to deduce log(R_ij_ + 1) values on the external conditions with acceptable accuracy. In addition, even the observed R_G_ values in the eight points external to the DOE resulted to be in good agreement with the predicted values. The maximum region in the R_G_ surface plots shown in Figure 3 identifies suitable combinations of T and φ for a given t_g_ level providing the best overall resolution in the chromatogram. Regarding the influence of t_g_, the surface shape is moderately dependent on this factor, but a t_g_ increase improves the separation according to a systematic up-shift of the surface. A decrease of t_g_ implies a steeper variation of the mobile phase composition during elution, which apparently does not facilitate separation of the saffron constituents. For t_g_ = 10 or 12 min a relatively wide region close to the lowest levels for both T and φ can be observed. The shape of the response surface suggests that the effects of T and φ on the resolution are not independent of each other. In particular, T influences the chromatogram resolution only at higher φ values, while this parameter has an almost negligible impact near the maxima of the surface plots. The optimal condition within the experimental domain was defined by the maximum level for t_g_ while T and φ value should be set to their minimum levels (t_g_ = 12 min, T = 25 °C and φ = 0.60 mL/min). To provide a final validation of the surface we collected a chromatogram close to this point (t_g_ = 10 min, T = 25 °C and φ = 0.65 mL/min). The observed R_G_ value in this point (0.320) was in good agreement with the predicted value (0.322). Because of small influence of t_g_ above the middle level for this factor, chromatograms collected at t_g_ = 12 min, T = 25 °C, φ = 0.60 mL/min and t_g_ = 10 min, T = 25 °C, φ = 0.60 did not show appreciable difference in terms of resolution. Therefore, t_g_ was finally set to 10 min. 

### 2.2. Ultra-High Performance Liquid Chromatography Saffron Analysis Using the Kinetex C18 Column

**Figure A1:**
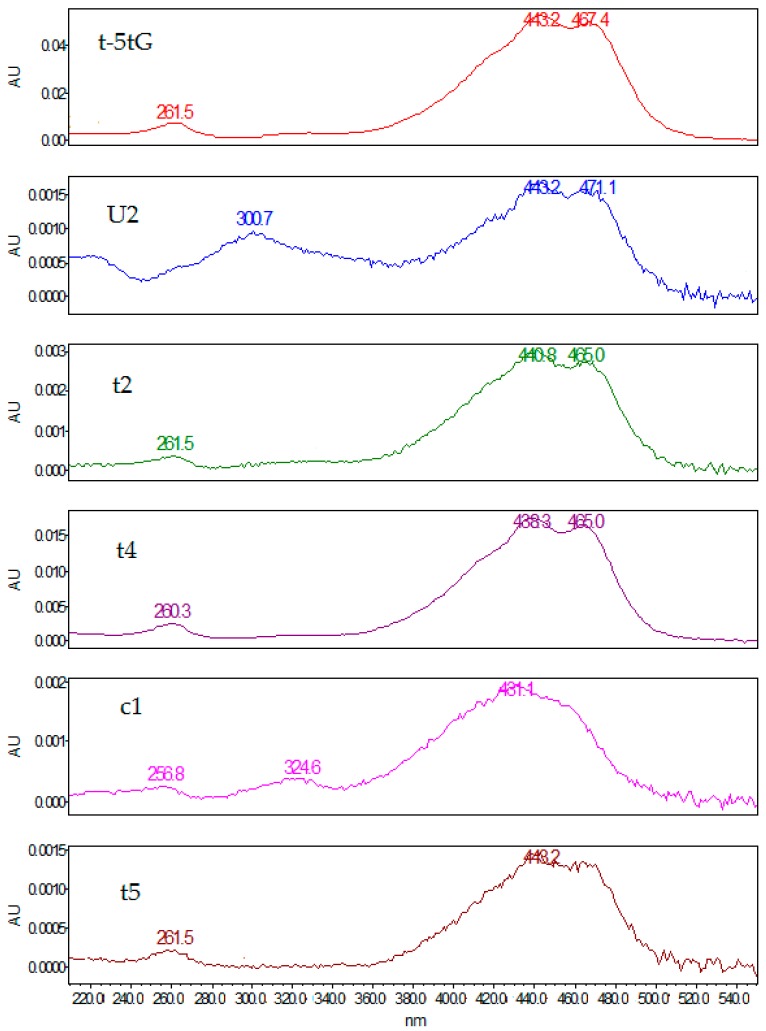
Ultraviolet-vis spectra of some compounds detected with the Kinetex C18 column (chromatogram A displayed in Figure 1).

In addition to the saffron coming from L’Aquila (Abruzzo, Italy), investigated in the ANN-based optimization stage, other two samples produced in Morocco and Iran in 2015 were analyzed in this work. All the three saffron samples were characterized by ultraviolet (UV)-vis spectrophotometry and resulted to belong to the best quality category (I) according to the ISO-3632 guidelines [31]. In particular, the observed $E_{1\mathrm{cm}}^{1\%}\left(440\;m\right)$ values quantifying the coloring strength were 254, 285 and 253, respectively. The chromatograms provided by the three saffron samples exhibited only moderate differences in the peak relative intensities. Moreover, water and water–methanol extracts showed very similar chromatograms. It follows that the formation of methyl esters of crocetin potentially caused by the presence of methanol in the extraction medium can be excluded. Figure 1A displays the observed chromatogram at the wavelength detection of 440 nm under the optimal separation conditions. Table 1 shows the retention times and a tentative qualitative identification of the detected solutes. The majority of the compounds with RT in the range 5–12 min shows the typical UV-vis spectrum of crocins, and the *cis* or *trans* isomeric form of crocetin was unequivocally defined. Figure A1 (Appendix A) displays the UV-vis spectra of some detected compounds. However, chemical structure of only the major crocins can be safely assigned based on the relative peak intensities and elution order reported in literature. Some detected compounds, U1–U4, although showing a strong absorption band in the 400–450 nm range typical of carotenoids, cannot be identified as crocins, because of a non-negligible shift in the maxima positions compared to the expected values. Moreover, low absorption intensity and noise in the 200–340 nm range did not enable accurate identification of secondary maxima that are diagnostic in qualitative identification. Nevertheless, 32 chromatographic peaks can be safely attributed to crocetin esters. In agreement with recent studies [13,14], this confirms that the number of crocetin derivatives occurring in the *Crocus sativus* L. stigmas is greater than those structurally characterized by means of HPLC-MS. Moreover, 21 out of 32 crocins are *trans*-crocetin derivatives. It is evident that such large number of derivatives cannot be originated by mono- and di-esterification of crocetin with only the four glucoside moieties identified so far, but additional sugars should be involved.

### 2.3. Ultra-High Performance Liquid Chromatography Saffron Analysis Using the Luna Omega Polar C18 Column

**Figure 4:**
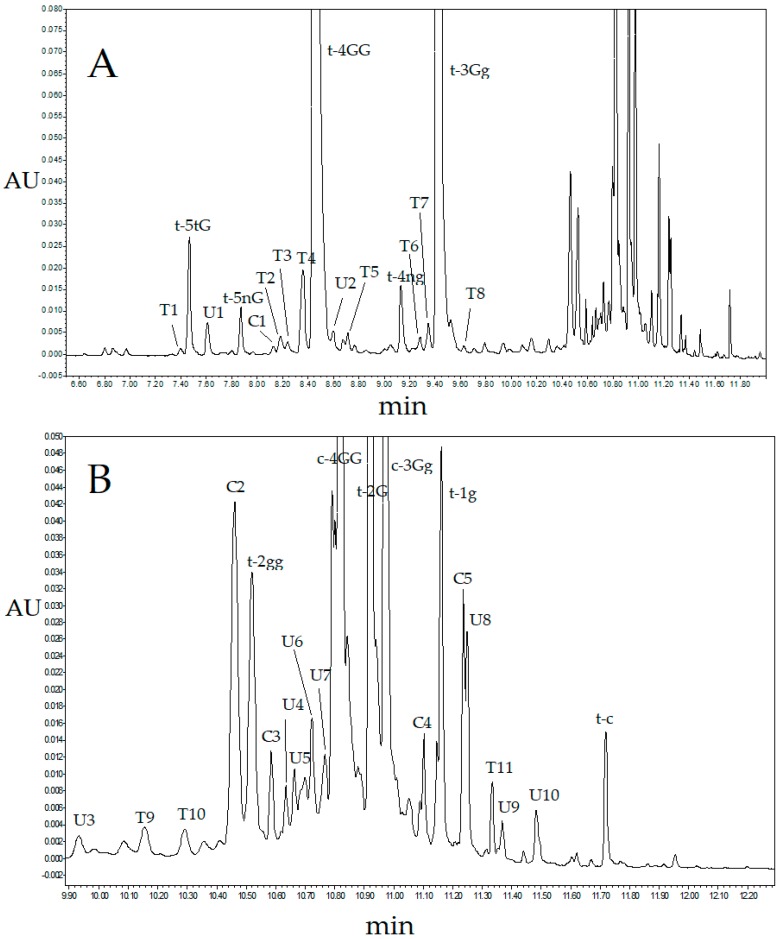
(**A**) Full UHPLC chromatogram detected at 440 nm of a saffron extract obtained with the column Luna Omega Polar C18 under the conditions described in Section 3.4 and (**B**) magnification of the last part of the chromatogram. Peak assignments are reported in Table 2.

To confirm the unexpectedly large number of detected crocetin derivatives, the saffron extracts were analyzed on a Luna Omega Polar C18 (Phenomenex) column packed with a novel stationary phase having a polar modified surface. Separation was conducted under the optimal conditions found for the Kinetex C18 (Phenomenex) column except for the eluent gradient slope that was slightly modified as described in Section 3.4. Figure 4 shows the observed chromatogram detected at 440 nm and Table 2 displays a tentative assignment of the observed peaks. Compared with the chromatograms collected with the Kinetex C18 column, the Luna Omega Polar C18 column provided a better separation of the less retained saffron components, although resolution was moderately worse at higher retention times. In particular, the intense peak eluting before the most abundant crocin t-4GG in the chromatogram collected with the first column (Figure 1) and assigned to t-5nG seems to split in a number of less intense peaks in the chromatogram provided by the latter (Figure 4). On the other hand, the most abundant *cis*-crocins c-4GG and c-3Gg and t-2G, that exhibit quite different retention times on the Kinetex C18, give rise to much closer peaks in the chromatogram collected with the Luna Omega Polar C18 column. In spite of the loss of resolution in this region of the chromatogram, 27 crocins, 24 of which deriving from *trans*-crocetin, were detected (Table 2). The contents of the major crocins in the analyzed saffron samples using both Kinetex C18 and Luna Omega Polar C18 columns, determined according to the procedure described in Section 3.5, are compared in Table 3 to literature data [32,33]. The concentrations of the three major crocins, t-4GG, t-3Gg and c-3Gg, taking into account of possible moderate fluctuations related with the saffron origin and aging, are comparable to that reported in literature. This result is not unexpected because the relatively large peak areas of these analytes can be accurately measured and possible co-elution with minor saffron metabolites does not alter much the observed area. On the other hand, co-elution of t-5nG with other saffron components in the chromatogram of Kinetex C18 column is presumably responsible of the higher estimated concentration of this compound as compared with literature data or the values obtained from the chromatogram provided by the Luna Omega Polar C18 column. Analogously, the non-ideal separation of the most retained saffron metabolites using the Luna Omega Polar C18 column could be responsible for the moderately higher concentrations estimated for c-4GG, c-3Gg and t-2G. Regardless of the kind of column used in this work and the saffron provenance, the estimated concentration of the crocin t-2gg is markedly lower than the value reported in literature. This can be due to the fact that both UHPLC columns were able to separate t-2gg and a *cis*-crocin eluting only just before (Figure 1 and Figure 4), while the two compounds may co-elute in HPLC analyses. In summary, despite the two UHPLC columns provided quite different chromatograms in terms of resolution, a larger number of crocetin esters than those normally observed in HPLC analysis were detected using both stationary phases. It should be remarked that the good performance provided by the Luna Omega Polar C18 column could be further improved by a careful tuning of the separation conditions; further work on this aspect is in progress. Preliminary mass fragmentation patterns acquired by coupling the UHPLC columns with MS detectors support the assignment of newly identified compounds to the family of crocetin esters, here based on the UV-vis spectra. In particular, using an electrospray ionization (ESI)-MS detector and a mobile phase acidified with formic acid (1%), MS spectra were recorded in the range of mass/charge ratio between 50 and 1200, both in the negative and positive ion mode. In the positive ion mode, the observed quasi-molecular ions were principally adducts with sodium and potassium. In negative ion mode detection, deprotonated quasi-molecular ions were identified. In both cases, the observed fragment ions were generated by the loss of glycosides. Based on these data, the following crocetin esters were identified: (i) four crocins with five glucose units, (ii) five crocins with four glucose units, (iii) four crocins with three glucose units, (iv) six crocins with two glucose units and (v) two mono-glucosyl crocetin esters. Further UHPLC-MS work is planned to clarify the chemical structure of these saffron constituents.

## 3. Materials and Methods

### 3.1. Samples, Chemicals and Solvents

Saffron samples in stigmas produced in L’Aquila (Abruzzo, Italy), Morocco (Taliouine) and Iran (Khorasan province) in 2015 were analyzed. The samples were obtained directly from producers or consortia to guarantee their geographical origin and authenticity. High performance liquid chromatography-grade methanol and acetonitrile were purchased from Sigma-Aldrich (St. Louis, MO, USA). Double deionized water was obtained from a Milli-Q filtration/purification system (Millipore, Bedford, MA, USA).

### 3.2. Saffron Characterization Using Ultraviolet-Vis Spectroscopy

Sample preparation was carried out according to the procedure ISO-3632 [31,34], but saffron and solvent amounts were reduced proportionally: 10 mg of grinded saffron were suspended in 20 mL volumetric flask filled with 18 mL of distilled water; the suspension was kept under magnetic stirring for 1 h in the dark and finally diluted to 20 mL. The spectrophotometric measurement was carried out on a suitable aliquot of aqueous extract after a 10-fold dilution and filtration on a 0.45 μm Whatman Spartan 13/0.2 regenerate cellulose filter (Whatman, GE Healthcare Life Sciences, Little Chalfont, UK). The UV-vis spectrum was acquired in the 200–700 nm range with a Cary 50 Probe (Agilent Technologies, Santa Clara, CA, USA) spectrophotometer using a 1 cm pathway quartz cuvette (Agilent Technologies) and pure water for blank correction. Moisture was determined by evaluating the weight loss after the saffron sample (100 mg) had kept in oven at 103 °C for 16 h.

### 3.3. Ultra-High Performance Liquid Chromatography Sample Preparation

About 100 mg of saffron stigmas were gently grinded in a mortar. Fifty mg of powdered sample were successively transferred into a 50 mL volumetric flask and extracted with a water–methanol 1:1 *v*/*v* mixture in the dark and under magnetic stirring for one hour. The extract was finally centrifuged at 140 g and filtered on 0.45 and 0.2 μm Whatman Spartan 13/0.2 RC cellulose filters (Whatman).

### 3.4. Ultra-High Performance Liquid Chromatography Analysis

The saffron extracts were analyzed by means of an Acquity H-Class UHPLC system (Waters, Milford, MA, USA) equipped with a quaternary solvent manager, a sample manager, a column heater, a photodiode array detector and a degassing system. Data handling was managed by Empower v.3.0 software (Waters). The mobile phase consisted of water (eluent A) and acetonitrile (eluent B) erogated according to the following gradient profile: 10% B to 45% B in a variable time t_g_ (between 8 and 12 min); 45% B to 90% B in 2 min; 90% B kept for 1 min; 90% B to the initial composition in 2 min and the column was re-equilibrated for 2 min. The eluent flow rate was varied between 0.6 and 1.0 mL/min. The saffron extracts (3 μL) were injected into the UHPLC system equipped with a Kinetex C18 (Phenomenex, Torrance, CA, USA) reversed-phase column with 100 mm length, 4.6 mm internal diameter and 2.6 μm particle size, protected by a C18 SecurityGuard ULTRA pre-column (Phenomenex). The column was termostated within the temperature range 25–35 °C while the samples were kept at 15 °C. Ultra-high performance liquid chromatography analyses of saffron extracts were also carried out using a Luna Omega Polar C18 (Phenomenex) column with 100 mm length, 2.1 mm internal diameter and 1.6 μm particle size. The column temperature was set to 25 °C and eluent flow rate to 0.6 mL/min. Starting from the optimal condition found for the Kinetex C18 column, the eluent gradient was slightly modified as follows: 5% B to 30% B in 10 min; 30% B to 90% B in 2 min; 90% B kept for 1 min; 90% B to the initial composition in 2 min. The column was re-equilibrated for 2 min before successive analysis.

### 3.5. Quantitative and Qualitative Analysis

Qualitative identification of the observed HPLC-DAD chromatographic peaks was attempted on the basis of peculiar and well-known absorption spectra of saffron constituents, together with the relative peak intensities and elution order in HPLC chromatograms described in literature for similar separation conditions [4,7,10]. Crocins show the characteristic UV-vis spectra of the carotenoid moiety of the molecules featured by a relatively strong double-peaks band at 400–500 nm. Both *trans*- and *cis*-crocins, apart from the intense band in the visible region, exhibit a secondary absorption at 260–264 nm and the *cis*-crocins alone display an additional relative maximum at 326–327 nm [4,10]. Since analytical standards of most saffron metabolites are lacking, a method based on the combination of HPLC-DAD peak areas observed at 440 nm with the extinction coefficients determined by spectroscopic measurements [32] was applied for quantification of individual crocins. The concentration of the crocin i was determined using the equation:

(1) $$c\left(\mathrm{mg}/g\right)=\frac{{\mathrm{Mw}}_{i}\cdot E_{1\mathrm{cm}}^{1\%}\left(440\;\mathrm{nm}\right)\cdot A_{i}}{\varepsilon_{t,c}},$$

where Mw_i_ and A_i_ are the molecular weight and the percentage peak area, respectively, $E_{1\mathrm{cm}}^{1\%}(440\;\mathrm{nm}$) is the coloring strength of the saffron sample and ε_t,c_ is the extinction coefficient (89,000 M^−1^cm^−1^ for *trans*-crocins and 63,350 M^−1^cm^−1^ for *cis*-crocins). 

### 3.6. Multivariate Design of Experiments and Statistical Data Treatment

#### 3.6.1. Design of Experiments

Design of experiments is a powerful statistical method to establish the relationship between factors affecting a process and the output of that process [30,35]. In optimization problems, DOE enables to design an informative set of experiments, varying together the levels of all the involved factors. As compared with univariate methods, in which only one factor is varied at a time, a larger experimental domain can be explored using a generally lower number of experiments and interactive effects among the variables can be investigated. Once the response to be optimized has been experimentally determined in the points of the selected DOE, application of a regression model to the experimental data allows predicting the value of the response in any point of the experimental domain. The polynomial regression is the most common approach to generate a response surface, but other different multivariate techniques, including ANN modeling, can be used in RSM [35]. While a polynomial function such as linear, first-order interaction or second-order quadratic, must be specifies in conventional RSM, ANN-modeling does not require the preliminary definition of a fitting equation. 

**Figure 5:**
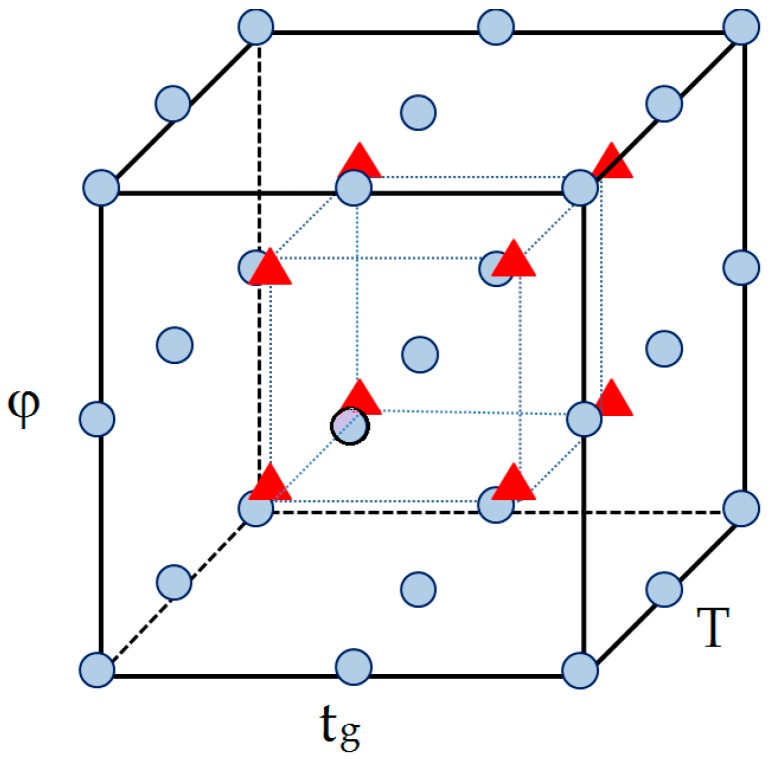
Three-level full factorial design used to optimize separation of the saffron components with the Kinetex C18 column. Variables and levels are given in Table 2. Circles and triangles identify experimental conditions used in calibration and prediction of the ANN-based response surface methodology (RSM) model for resolution, respectively.

In this work, we evaluated the combined effect of the eluent flow rate (φ), the column temperature (T) and the duration of the first linear step of the eluent gradient profile (t_g_) on the chromatogram resolution. A full-factorial three-level DOE [35] was used to identify suitable combinations of the above three factors. Table 4 displays the three levels, lower, medium and higher (−1, 0 and 1, respectively), for each factor. Eight additional experiments were performed in the central points of the eight cubic subspaces of the experimental domain and were used for external validation. Figure 5 graphically displays the points of the full-factorial DOE and the external data points. 

The chromatograms collected according to the DOE were evaluated to identify the peaks to be considered in multivariate analysis. We did not consider isolated signals, but we focused our attention on close peak pairs or partially overlapped signals whose resolution was expected to improve by tuning of the experimental conditions. Finally, 22 chromatographic peaks were selected. For each collected chromatogram, the individual resolution of adjacent peaks R_ij_ was determined according to the following relationship:

R_ij_ = 2 (RT(j) − RT(i)/(W_1/2_(i) + W_1/2_(j)), (2)

where RT(j) and RT(i) are the retention times of the second and the first peak of the pair, and W_1/2_(i) and W_1/2_(j) are the peak widths.

#### 3.6.2. Artificial Neural Network Modeling

Three-layer feed-forward ANNs [36,37] were used in this work. The single processing units, neurons, are organized in three layers: one input layer that collects the independent variables, one output neuron providing the network response and one hidden layer with an adjustable number of neurons fully connected to both input and output neurons. Weights associated to the connections modulate information flowing from the input layer to the output neuron. The weighted signals coming from the input neurons entering each hidden neuron are summed, added to a bias value (equivalent to a weight associated to an input signal 1) and the result is transformed by a non-linear activation function providing an output signal. The output neuron operates on the weighted outputs of the hidden neurons performing a similar computation, which gives the ANN final answer. Artificial neural network-based model calibration consists in a training procedure based on the iterative adjustment of weights to produce the best agreement between target and computed responses for a suitable number of input/output pairs (training or learning set). The optimized weights, which represent a sort of ANN memory, can be recalled to deduce the response if the predictors are known. A quasi-Newton method [37] that incorporates second order information about the shape of error surface was used here to train the network. To avoid overfitting, due to incorporation of the noise present in the training data and the subsequent loss of generalization capability, the ANN performance was tested on a validation data set after each learning epoch. The input and output variables were subjected to range-scaling between 0 and 1 and a linear activation function was always applied in the output neuron. Minimum of validation error was the criterion adopted to stop learning and to select, among alternative trained networks differing in the topology and kind of the activation function (logistic or hyperbolic tangent) in the hidden neurons, the one with the best prediction ability. Several methods have been proposed to assess the contributions of the input variables to the network response [28]. In this work, sensitivity analysis was performed by partial derivative method based on the computation of the first partial derivative of the output with respect to a particular input. The performance of the ANN-based model in the different points of the DOE was evaluated by SRD developed by Héberger [29] to compare models or methods. The ranking differences are calculated as Euclidian distances between the ranking of observations of models/methods and a reference ranking, and subsequently summed. Comparison of ranks with random numbers can be used to validate SRD analysis. Software OpenNN [38] was used to perform ANN regression. 

## 4. Conclusions

In this work, an artificial neural network was successfully used to model the influence of the column temperature, the eluent flow rate and the slope of a linear eluent concentration gradient on the UHPLC separation of the saffron water-soluble components. A sensitivity analysis based on the partial derivative method revealed that the above three experimental factors have a comparable importance to define the resolution of close chromatographic peaks. Generalisation ability of the ANN-based model was demonstrated using an external prediction set. The substantial equivalent performance of the ANN-based model in different experimental conditions was also confirmed by SRD method. Moreover, this investigation revealed that the family of crocetin esters, the main coloured constituents of saffron, consists of a greater number of derivatives than those structurally characterised so far by MS data and usually detected in HPLC analysis. The better performance provided by UHPLC combined with chemometrics-based optimisation of separation allowed to both reduce peak overlapping and detect new minor crocetin derivatives. The large number of crocins observed in the UHPLC chromatograms based on a conventional reversed-phase C18 column was confirmed by the chromatographic data collected with a novel UHPLC stationary phase applied to saffron samples produced in different countries. It follows that further sugar moieties should be involved in the formation of crocetin esters in addition to those previously identified (glucoside, gentiobioside, neapolitanoside and triglucoside). Preliminary mass-spectrometry data supports the assignment of the newly found saffron components to the class of crocetin derivatives and further work is in progress to clarify their chemical structure.

## Figures and Tables

**Table 1:** List of the saffron metabolites found in the UHPLC-DAD chromatograms detected at 440 nm collected with the column Kinetex C18: retention time (RT) observed under application of the optimal separation conditions, assigned structure or chemical class, absolute and relative maxima of the absorption spectra.

RT (min)	Compound/Chemical Class	Abbreviation ^a^	λ_max_
5.34	unknown	U1	230, 423
5.59	*trans*-crocetin (tri-β-d-glucosyl)(β-d-gentiobiosyl) ester	t-5tG ^b^	262, 443, 466
5.91	*trans*-crocin	t1 ^b^	262, 441, 465
5.99	*trans*-crocetin (β-d-neapolitanosyl)(β-d-gentiobiosyl) ester	t-5nG ^b^	241, 418, 440
6.20	unknown	U2	301, 443, 471
6.28	*trans*-crocin	t2	262, 442, 466
6.37	*trans*-crocetin bis(β-d-gentiobiosyl) ester	t-4GG ^b^	262, 441, 465
6.53	*trans*-crocin	t3 ^b^	259, 438, 465
6.60	*trans*-crocin	t4 ^b^	260, 440, 465
6.72	*cis*-crocin	c1 ^b^	256, 325, 431, 440 sh
6.83	*trans*-crocetin (β-d-neapolitanosyl)(β-d-glucosyl) ester	t-4ng ^b^	261, 441, 464
7.00	*trans*-crocin	t5	261, 438, 464
7.05	*trans*-crocin	t6	260, 441, 463
7.13	*trans*-crocetin (β-d-gentiobiosyl)(β-d-glucosyl) ester	t-3Gg ^b^	262, 441, 465
7.30	*trans*-crocin	t7 ^b^	260, 440, 463
7.43	unknown	U3 ^b^	317, 426, 444 sh
7.63	*trans*-crocin	t8 ^b^	260, 441, 466
7.70	*trans*-crocin	t9 ^b^	253, 440, 463
7.83	unknown	U4 ^b^	243, 308, 413, 438sh
7.91	*cis*-crocin	c2 ^b^	263, 329, 440, 465
8.04	*trans*-crocetin bis(β-d-glucosyl) ester	t-2gg ^b^	260, 440, 465
8.46	*trans*-crocin	t10 ^b^	258, 435, 460 sh
8.56	*trans*-crocin	t11 ^b^	252, 432, 462 sh
8.66	*cis*-crocin	c3 ^b^	223, 265, 327, 439
8.75	*cis*-crocin	c4	262, 329, 440, 463
8.87	*cis*-crocetin bis(β-d-gentiobiosyl) ester	c-4GG ^b^	262, 326, 435, 458 sh
9.06	*trans*-crocin	t12	264, 442, 467
9.65	*cis*-crocin	c5 ^b^	262, 327, 434, 457
9.70	*cis*-crocetin (β-d-gentiobiosyl)(β-d-glucosyl) ester	c-3Gg ^b^	263, 326, 433, 460
10.66	*cis*-crocin	c6	263, 326, 432, 456 sh
11.06	*trans*-crocin	t13	263, 443, 469
11.76	*trans*-crocetin mono(β-d-gentiobiosyl) ester	t-2G	258, 434, 459
11.85	*trans*-crocin	t14	259, 431, 455
11.90	*cis*-crocetin bis(β-d-glucosyl) ester	c-2gg	321, 426, 451
11.94	*cis*-crocetin mono(β-d-gentiobiosyl) ester	c-2G	258, 313, 426, 450 sh
12.22	*trans*-crocetin mono(β-d-glucosyl) ester	t-1g	257, 432, 458

^a^ Abbreviation in nomenclature of known crocins was adopted from reference [4]; ^b^ chromatographic peaks considered in artificial neural network (ANN) modeling.

**Table 2:** List of the saffron metabolites found in the UHPLC-DAD chromatograms detected at 440 nm collected with the Luna Omega Polar C18 column under the conditions described in Section 3.4: retention time (RT), assigned structure or chemical class, absolute and relative maxima of the absorption spectra.

RT (min)	Compound/Chemical Class	Abbreviation ^a^	λ_max_
7.40	*trans*-crocin	T1	259, 444, 466
7.46	*trans*-crocetin (tri-β-d-glucosyl)(β-d-gentiobiosyl) ester	t-5tG	262, 442, 465
7.60	unknown	U1	243, 418, 440
7.86	*trans*-crocetin (β-d-neapolitanosyl) (β-d-gentiobiosyl) ester	t-5nG	262, 440, 464
8.16	*cis*-crocin	C1	260, 327, 441, 464
8.20	*trans*-crocin	T2	262, 441, 462 sh
8.26	*trans*-crocin	T3	259, 442, 464
8.37	*trans*-crocin	T4	264, 437, 466 sh
8.48	*trans*-crocetin bis(β-d-gentiobiosyl) ester	t-4GG	262, 443, 466
8.60	unknown	U2	244, 415, 440
8.71	*trans*-crocin	T5	262, 437, 464
9.14	*trans*-crocetin (β-d-neapolitanosyl)(β-d-glucosyl) ester	t-4ng	262, 441, 465
9.28	*trans*-crocin	T6	261, 438, 464
9.35	*trans*-crocin	T7	260, 441, 463
9.44	*trans*-crocetin (β-d-gentiobiosyl)(β-d-glucosyl) ester	t-3Gg	262, 441, 465
9.52	*trans*-crocin	T8	260, 440, 464
9.94	unknown	U3	308, 413, 434
10.16	*trans*-crocin	T9	262, 442, 464
10.30	*trans*-crocin	T10	262, 440, 461 sh
10.46	*cis*-crocin	C2	263, 329, 440, 465 sh
10.53	*trans*-crocetin bis(β-d-glucosyl) ester	t-2gg	262, 327, 440, 465
10.58	*cis*-crocin	C3	262, 315, 442, 466
10.67	unknown	U4	239, 409, 434
10.71	unknown	U5	245, 321, 440, 465
10.77	unknown	U6	254, 301, 434, 466 sh
10.80	unknown	U7	329, 487
10.85	*cis*-crocetin bis(β-d-gentiobiosyl) ester	c-4GG	262, 326, 434, 458
10.92	*trans*-crocetin mono(β-d-gentiobiosyl) ester	t-2G	258, 435, 459
10.90	*cis*-crocetin (β-d-gentiobiosyl)(β-d-glucosyl) ester	c-3Gg	262, 326, 434, 460
11.09	*cis*-crocin	C4	258, 323, 432, 458
11.15	*trans*-crocetin mono(β-d-gentiobiosyl) ester	t-2G	258, 435, 459
11.23	*cis*-crocin	C5	320, 428, 451
11.25	unknown	U8	428, 451
11.32	*trans*-crocin	T11	257, 434, 458
11.36	unknown	U9	234, 402, 427
11.47	unknown	U10	264, 442, 467
11.71	*trans*-crocetin	t-c	258, 433, 458

^a^ Abbreviation in nomenclature of known crocins was adopted from ref [4].

**Table 3:** Concentration (mg/g) of the major crocins found in the saffron samples analyzed in this work by UHPLC-DAD; average values and standard errors obtained in triplicate experiments and comparison with literature data.

Crocin	Column				Ref [33]	Ref [32]
	Kinetex C18	Luna Omega Polar C18				
	Sample					
	AQ	AQ	IR	MO		
t-5tG	3.32 ± 0.06	2.8 ± 0.6	3.1 ± 0.2	3.1 ± 0.4	3.6 ± 0.1	
t-5nG	13.6 ± 0.1	0.8 ± 0.1	1.09 ± 0.09	0.9 ± 0.1	3.8 ± 0.1	
t-4GG	159.8 ± 0.4	153 ± 1	146.9 ± 0.8	169 ± 1	157.2 ± 0.3	145 ± 3
t-3Gg	54.5 ± 0.4	52.3 ± 0.1	50.8 ± 0.1	60.4 ± 0.5	76.4 ± 0.4	70 ± 2
t-2gg	2.46 ± 0.06	1.84 ± 0.08	1.76 ± 0.08	2.12 ± 0.09	6.0 ± 0.2	
c-4GG	11.5 ± 0.2	19.6 ± 0.4	19.2 ± 0.8	26 ± 1	4.8 ± 0.3	12 ± 1
c-3Gg	2.13 ± 0.08	8.0 ± 0.6	5.0 ± 0.3	7.2 ± 0.6	2.4 ± 0.1	5.2 ± 0.4
t-2G	4.0 ± 0.1	9.6 ± 0.8	12.2 ± 0.2	11.4 ± 0.6	9.8 ± 0.2	4.8 ± 0.2
t-1g	0.47 ± 0.02	0.77 ± 0.09	1.37 ± 0.04	0.83 ± 0.09	0.9 ± 0.1	

AQ, L’Aquila (Italy); IR, Iran; MO, Morocco.

**Table 4:** Factors and levels of the full-factorial design of experiments (DOE).

Factors	Level		
	−1	0	1
T (°C)	25	30	35
t_g_ (min)	8	10	12
φ (mL/min)	0.6	0.8	1.0
